# Low-dose Bacillus Calmette-Guerin versus full-dose for intermediate and high-risk of non-muscle invasive bladder cancer: a Markov model

**DOI:** 10.1186/s12885-018-4988-z

**Published:** 2018-11-12

**Authors:** Zongren Wang, Han Xiao, Guangyan Wei, Ning Zhang, Mengchao Wei, Zebin Chen, Zhenwei Peng, Sui Peng, Shaopeng Qiu, Heping Li, Jianting Long

**Affiliations:** 1 Department of Urology, The First Affiliated Hospital of Sun Yat-Sen University, Guangzhou, Guangdong China; 2 Department of Gastroenterology and Hepatology, The First Affiliated Hospital of Sun Yat-Sen University, Guangzhou, Guangdong China; 3 Department of Liver Surgery, The First Affiliated Hospital of Sun Yat-sen University, Guangzhou, Guangdong China; 4 Department of Clinical Trials Unit, The First Affiliated Hospital of Sun Yat-Sen University, Guangzhou, Guangdong China; 5 Department of Oncology, The First Affiliated Hospital of Sun Yat-sen University, Guangzhou, Guangdong China

**Keywords:** Bladder cancer, BCG, Intravesical therapy, Markov model

## Abstract

**Background:**

To compare the efficacy of low dose (27 mg) Bacillus Calmette-Guérin (BCG) and a full dose (81 mg) BCG immunotherapy for patients with intermediate and high-risk non-muscle invasive bladder cancer (NMIBC) after a typical transurethral bladder resection.

**Methods:**

We constructed a Markov model for a 20-year simulation of the disease to compare the overall survival of patients with intermediate and high-risk of NMIBC between the full-dose therapy (FD group) and the low-dose therapy (LD group). Base case analysis, one-way and two-way sensitivity analysis and a second-order Monte Carlo analysis were performed based on data from 15 published articles.

**Results:**

The expected overall survivals were 9.56 (9.55–9.57) years for FD group and 9.63 (9.61–9.64) years for LD group(*P* < 0.001). The estimated mortality in the FD group at 5, 10, and 20 years were 34.23%, 57.51% and 83.14%, respectively. The corresponding values in the LD group were 34.11%, 57.17%, 82.16%, respectively. Age-specific mortality and metastatic rate after undergoing radical cystectomy (RC) were the most two sensitive parameters in both groups. The rate of disease recurrence with disease worsening is the determining factor when choosing the optimal dose of BCG treatment.

**Conclusions:**

A low-dose BCG treatment may act slightly better than a full-dose BCG treatment for patients with intermediate and high-risk of NMIBC. This finding will require further high-quality studies to validate.

**Electronic supplementary material:**

The online version of this article (10.1186/s12885-018-4988-z) contains supplementary material, which is available to authorized users.

## Introduction

Bladder cancer is known as the most common malignancy of the urinary tract [1]. About 75%~ 85% of the patients suffered from a non-muscle invasive bladder cancer (NMIBC) [2]. Even though the tumor can be completely resected by a transurethral bladder resection (TUR), the patients with NMIBC still experience a high recurrence rate. Accordingly, an intravesical Bacillus Calmette-Guérin (BCG) immunotherapy after initial TUR was reported to reduce the median time to recurrence from 38 to 22 months compared with TUR alone [3], and further studies also proved the significant advantage of BCG treatment [4, 5]. Therefore, current guideline recommends an adjuvant intravesical BCG immunotherapy for intermediate and high-risk NMIBC [1].

However, the toxicity and high costs of BCG are remarkable disadvantages leading to intolerance and interruption of BCG treatments. Bladder cancer represents the most expensive malignancy to manage and treat [6], especially for NMIBC patients who are in a rather high risk of recurrence that require multiple times of treatments. Reducing the medical costs for NMIBC patients has surely become a tough task for all the urologists. Moreover, a randomized trial published in 2000 showed that 14.8% of the patients treated with full dose of BCG had experienced local side effects, and 9.4% stop the treatment because of toxicity [7]. In order to reduce the treatment costs and the incidence of side effects, some studies started to focus on reducing the dose of BCG without compromising the efficacy. Several clinical trials have proved that low dose of BCG can be successfully used in the intermediate and high-risk NMIBC patients, with similar recurrence-free survival and progression-free survival, but a significant reduced toxicity [8–12]. In 2007, Antonio Ojea and others made a comparison between two groups of low-dose BCG treatments (27 mg for low dose group, 13.5 mg for very low group) for NMIBC patients. The results of this trial indicated that one third of the standard dose, BCG 27 mg, seemed to be the minimum effective dose [13]. Guidelines for superficial bladder cancer did not give an exact optimal dose for BCG treatment until 2013 when a large randomized controlled trial was performed. This trial randomized patients into 4 groups: one-third dose of BCG with a 1-year maintenance (1/3D-1 yr), full dose of BCG with a 1-year maintenance (FD-1 yr), one-third dose BCG with a 3-year maintenance (1/3D-3 yr) and full dose BCG with a 3-year of maintenance (FD-3 yr), and then compared their 5-year disease-free rate, time to progression and overall survival. The author came to a conclusion that intermediate-risk patients should be treated with FD-1 yr. while FD-3 yr. treatment is rather proper for high-risk patients [2], which was later quoted and recommended by the EAU guidelines in 2013 [14]. However, according to the results of the 4 groups, a 1/3D of BCG was not inferior to an FD in either primary or secondary objectives when they had the same maintenance duration, making the conclusion of this study less convincing. Later on, several meta-analyses focused again on comparing low dose BCG and full dose BCG. Some declared a remarkable reduced efficacy in the low dose BCG group [15, 16]. Another 2 meta analyses including more researches concluded no significant difference between the 2 groups [17, 18], leaving the issue still controversial. The Markov model is capable of estimating a disease’s outcome by simulating disease progression where patients move through different health states over the preset cycles. It has been successfully used in the simulation of a head-to-head comparison of treatment efficacy [19–22]. In this article, we constructed a Markov model to make a comparison of efficacy between full dose BCG treatment and one-third dose BCG treatment for patients with intermediate and high-risk NMIBC.

## Materials and methods

### Model construction

**Fig. 1:**
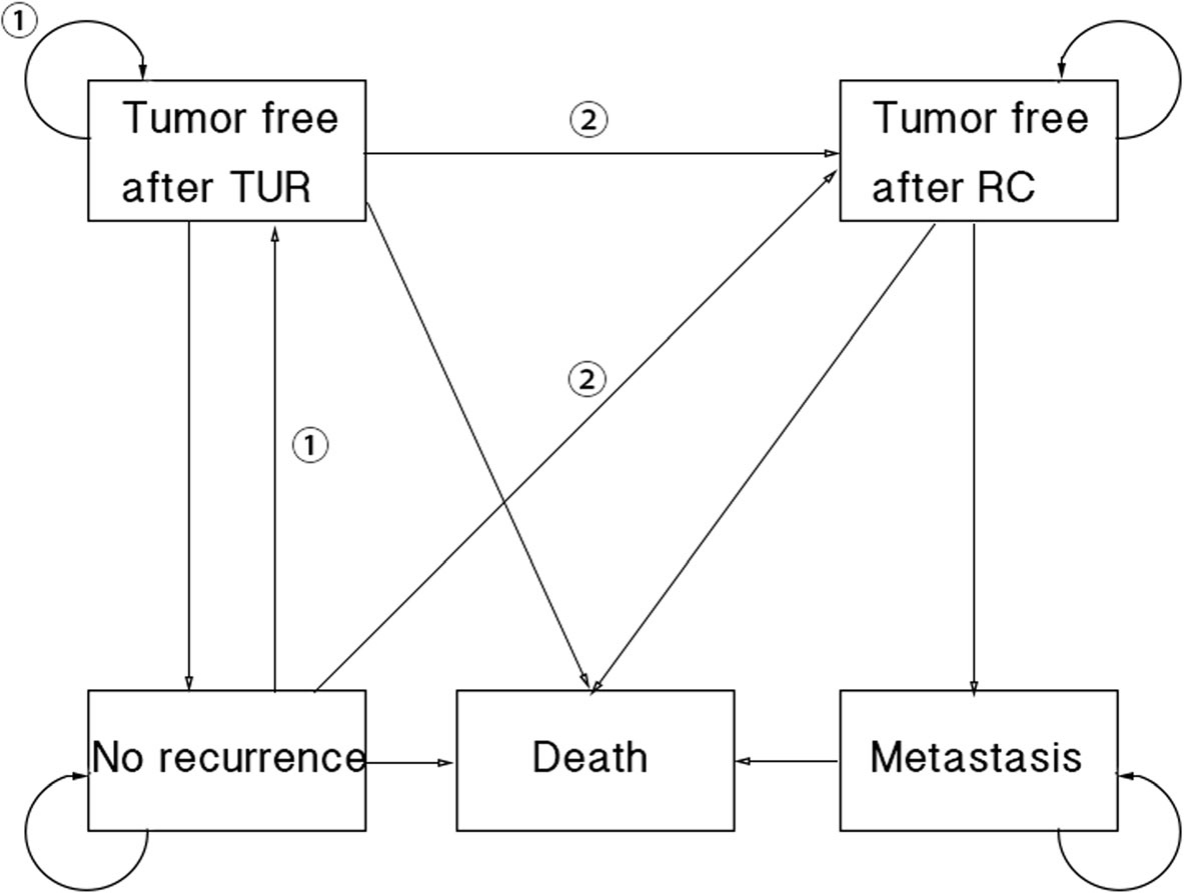
Flow diagram of Markov cohort model. Each pane represents a state of health. Straight lines with arrows indicate transition from one state to another while circular arrows stand for patients staying in the same state for more than one cycle. All the patients were supposed to be in the state of tumor free after TUR at the very beginning. Those without disease recurrence transform to the state of no recurrence. Those who experience disease recurrence without worsening of the disease would take another TUR surgery and the following BCG treatment and stay or transform into the state of tumor free after TUR (a), while those with worsening of the disease would take an RC surgery and transform to the state of tumor free after RC (b)

A multi-state Markov model is constructed to compare the treatment efficacy between full-dose intravesical BCG treatment (FD group) and low-dose intravesical BCG treatment (LD group) among intermediate and high-risk NMIBC patients. Although lots of randomized controlled trials (RCT) has been reported in this area, few of the follow-up periods are long enough to evaluate the whole course of NMIBC due to its low mortality. Since that the average age of researches cited in this article were 63.5–67.8 years, total follow up duration was set to be 20 years considering of the actual life expectancy for NMIBC patients. The terminal state was death, and the endpoint was overall survival in our study. Details of the model were represented in Fig. 1.

This model is a health state transition model initiated after a TUR surgery of intermediate and high-risk NMIBC patients (using the same risk group criterion with the EAU guidelines [14]). Two therapeutic decisions were designed as a 81 mg BCG intravesical therapy and a 27 mg BCG intravesical therapy after performing TUR, both including maintenance therapies for one year. The cycle length was 1 year, and the half-cycle correction was used [23]. The median survival, death within the 5-year, 10-year and 20-year period were measured in this model for all patients. The model comprised the following states: 1) tumor free after TUR (disease recurrence without worsening of the disease); 2) no recurrence (patients without disease recurrence); 3) tumor free after radical cystectomy (RC) (worsening of the disease requiring radical cystectomy); 4) metastasis (metastasis of the disease that cannot be treated by surgery); 5) death. Death is an absorbing state. Patients were allowed to stay in the same Markov state for more than 1 cycle. The annual state transition rates were derived from median survival or cumulative probability of survival (recurrence or progression) with the declining exponential approximation of life expectancy (DEALE) method [24]. The model was built by the TreeAge-Pro-2008 software (TreeAge Software Inc., Williamstown, MA, USA).

### Literature selection

All transition probabilities were obtained from the literatures published in English, retrieved from Pubmed with the latest searching on June 26, 2016. The following search terms were used: “Bacillus Calmette-Guérin” or “BCG”, “bladder cancer”, “radical cystectomy”, “death rate” or “mortality” and “metastasis”. Reference lists of the included studies were hand-searched to identify further relevant trials.

### Summary of transition probabilities and assumptions

We assembled data for calculating our transition probabilities from 7 RCTs, 2 prospective cohort studies, 4 retrospective studies and 2 literature reviews as showed in Table 1.

**Table 1:** Base Case Value and Sensitivity Range Used in the Model

• Transition probabilities	• Yearly base-case value (%)	• Range (%)
• Age-specific mortality	• 7.91 [27]	• –
• Rate of disease recurrence without worsening of the disease (LD group)	• 4.7 [10, 13] [25, 30]	• 3.99 [13]~ 7.05 [10]
• Rate of disease recurrence with worsening of the disease(LD group)	• 2.7 [10, 13] [25, 30]	• 2.06 [13]~ 5.98 [10]
• Rate of disease recurrence without worsening of the disease(FD group)	• 3.3 [4] [10, 26, 30]	• 1.55 [26] ~ 4.92 [4]
• Rate of disease recurrence with worsening of the disease(FD group)	• 3.7 [4] [10, 26, 30]	• 2.35 [26]~ 5.77 [10]
• Mortality of RC surgery	• 1.9 [31–33]	• 1 [33] ~ 5.2 [34]
• Metastatic rate after underwent RC	• 2.6 [35, 36]	• 2.3 [36] ~ 7.97[35]
• Death rate of metastatic state	• 44.1 [37]	• 42.99 [37] ~ 50 [38]

We designed our model based on the EAU guideline updated in 2013, and several assumptions were made as follows. Patients with disease recurrence without worsening of the disease were supposed to receive another TUR and then a following BCG treatment, while those with worsening of the disease were supposed to receive an RC. Patients underwent an RC were supposed to experience a risk of disease metastasis. According to the guideline, a 1-year maintenance of BCG treatment was recommended for intermediate risk group while a 1 to 3 years maintenance was recommended for high risk group. Focusing on comparison in dose of BCG, we set the maintenance period as one year for both group since that several early studies demonstrated a similar results of different maintenance period [2, 25, 26]. Analysis was also performed with a 3-year maintenance for both group. Results were included in Supplementary materials. An age-specific mortality was introduced into this model for disease-free patients (including Markov states of tumor free after TUR, tumor free after RC and no recurrence). With the mean age of patients involved ranging from 63.5 years to 67.8 years, we assumed the age-related mortality in this cohort as 0.0791 [27].

## Results

### Survival outcomes of the model

**Fig. 2:**
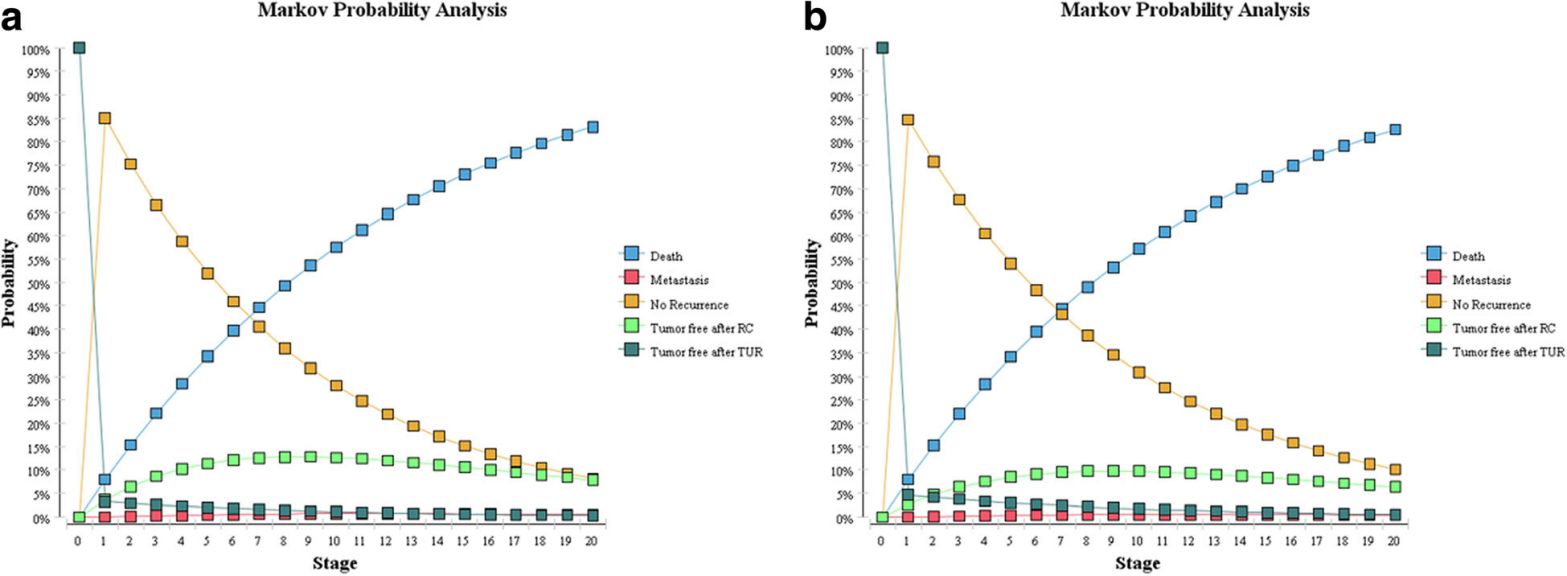
Markov probability analyses for the FD group (**a**) and the LD group (**b**).These diagrams showed the details of the distribution of the patients in each stage. The probability of no recurrence was higher in the LD group, while the probability of tumor free after RC was higher in the FD group. Abbreviations: FD, full-dose; LD, low-does; TUR, transurethral bladder resection; RC, radical cystectomy

**Fig. 3:**
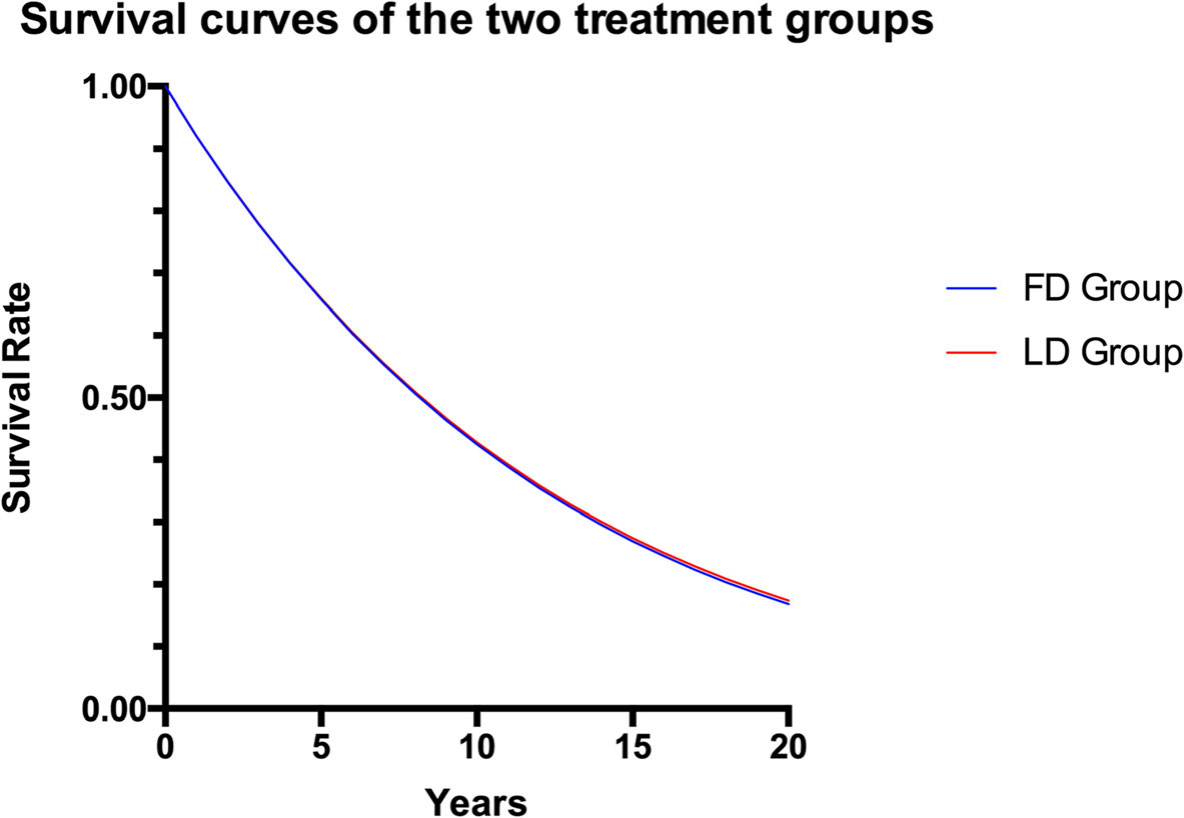
Survival curves of the two treatment groups. The two curves representing the survival rates of the FD group and the LD group were highly coincident, indicating that the priority of the LD group was not apparent. Abbreviations: FD, full-dose; LD, low-does

The expected overall survivals were 9.55 years for the FD group and 9.61 years for the LD group. The estimated mortality in the FD group at 5, 10, and 20 years were 34.23%, 57.51% and 83.14% respectively. The corresponding values in the LD group were 34.11%, 57.17% and 82.61%, respectively. The Markov probability analysis curves, which represented the distribution of patients among Markov states, indicated a higher proportion of the no recurrence state in the LD group (Fig. 2). The two survival curves are highly coincident as showed in Fig. 3.

### One-way and two-way sensitivity analysis

**Fig. 4:**
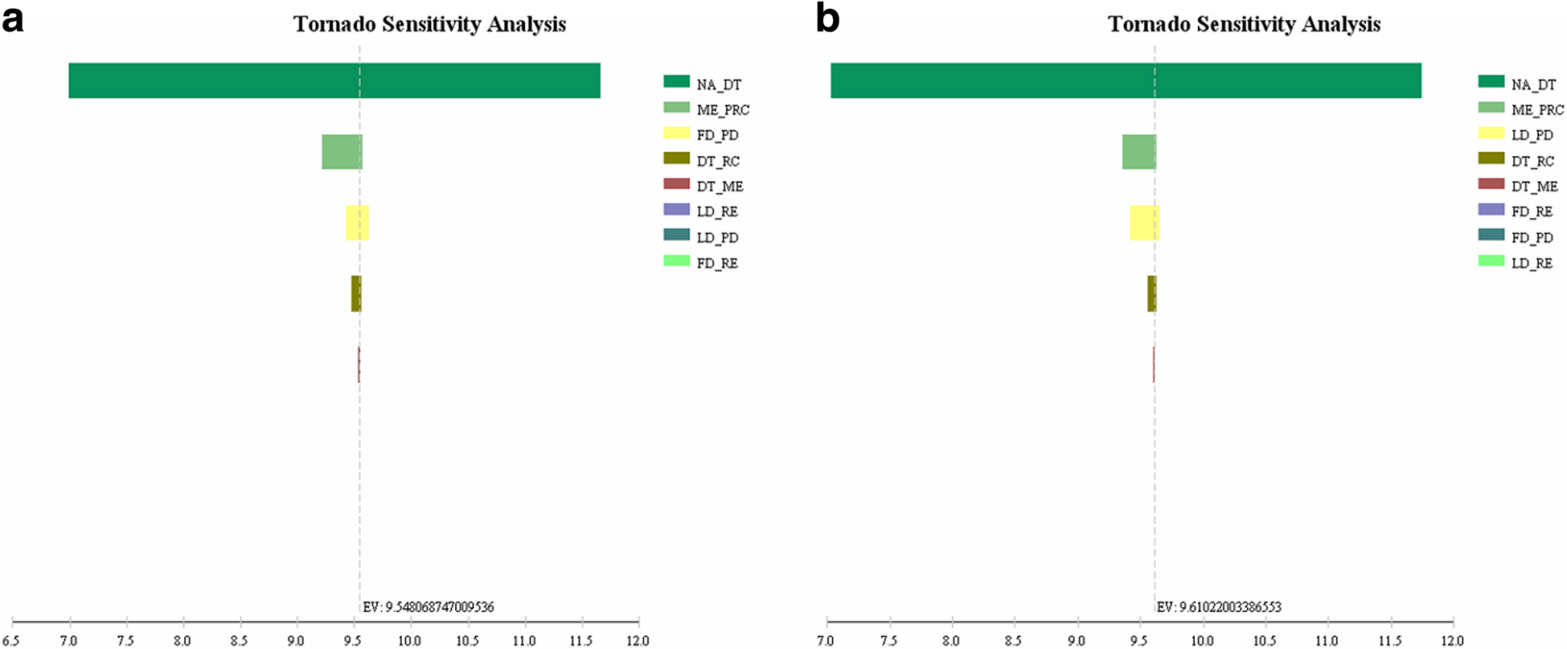
Tornado diagrams for the FD group (**a**) and the LD group (**b**). Tornado diagrams analyzed all the parameters in this model. The length of colored bar for each parameter represents the extent of its effect on the overall survival. The longer a bar is, the larger its effect is. Abbreviations: NA_DT, age-specific mortality; ME_PRC, metastatic rate after underwent RC; FD_PD, rate of disease recurrence with worsening of the disease(FD group); DT_RC, mortality of RC surgery; DT_ME, death rate of metastatic state; LD_RE, rate of disease recurrence without worsening of the disease (LD group); LD_PD, rate of disease recurrence with worsening of the disease(LD group); FD_RE, rate of disease recurrence without worsening of the disease(FD group)

Tornado diagrams analyzing the two therapies were showed in Fig. 4. Three most sensitive parameters for the LD group were age-specific mortality, metastatic rate after undergoing RC and rate of disease recurrence with disease progression. And for the FD group, they were age-specific mortality, metastatic rate after undergoing RC and rate of disease recurrence with disease progression. One-way analysis showed that the rate of disease recurrence with worsening of the disease seemed to affect the efficacy superiority between the two groups. The FD group would have a better efficacy if this rate of FD group was less than 2.7% (Additional file 1: Figure S1 (a)), or the rate of LD group was over 3.71% (Additional file 1: Figure S1(b)). All other parameters showed no effect on the superiority of LD group. A two-way sensitivity analysis of the two determinant parameters was performed (Additional file 1: Figure S2). An increase of the probability of disease recurrence with worsening of the disease reduced the efficacy superiority, which were similar in both groups.

### Second-order Monte Carlo simulation

**Fig. 5:**
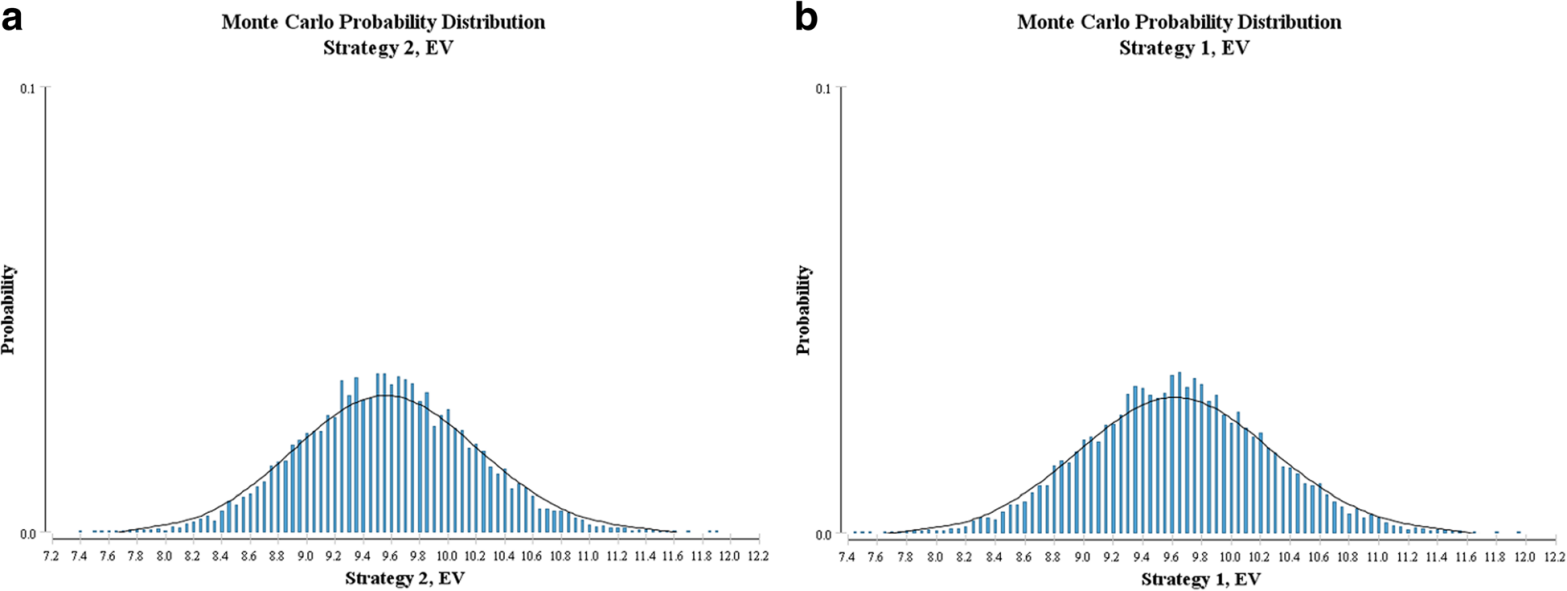
The second-order Monte Carlo probability distribution of the overall survival in the FD group (**a**) and the LD group (**b**). EV stands for expected value, which is the overall survival in this situation. The length of the bar represents the probability of an expected value in the 10,000 population. Abbreviations: EV, expected value

We ran a Monte Carlo simulation with the sample of 10,000 patients for each therapy, and details were showed in Fig. 5. The median survival of the FD group was 9.56 years compared to 9.63 years for the LD group. The 95%CI of overall survival in the FD group was (9.55–9.57) years, while that in the LD group was (9.61–9.64) years (*P* < 0.001), indicating a better but not apparent (compared to the expected OS of NMIBC patients) outcome in the LD group.

## Discussion

The optimal dose of intravesical BCG treatment for intermediate and high-risk NMIBC patients is still controversial to date. The original purpose to investigate on adjuvant dose of BCG was to reduce the costs and side effects of the treatment without affecting the efficacy, which might help explore a better cost-effective therapy and guide the treatment if with powerful evidences. However, conclusions of multiple studies were such diverse from each other that there seemed no conclusion could be commonly accepted. Therefore, further studies and new research methods could be meaningful to this unsolved problem. The Markov model mentioned in this article is generally used in medical decision making these years [19–22]. A Markov model arranges patients in different health states in the corresponding Markov states according to the natural disease history. Patients are supposed to be in one of those states and to transform from one to another in the next cycle (or stay the same). And based on the transition probabilities, the Markov model is able to simulate the process and outcomes of the disease by loop computation. It is particularly suitable for a decision problem involving a risk that is ongoing over time [28], which, in this case, stood for the remarkable incidence of recurrence and progression in NMIBC patients who were tumor free after a TUR. Thus, a Markov model was considered to be proper for the decision making of BCG treatments in NMIBC patients.

Our results showed that the overall survival of the LD group was better than that of the FD group. This consequence did not concur to out presupposition but similar results can be found in earlier studies. An RCT performed on intermediate and high-risk NMIBC patients by Oddens, et al. in 2013 showed that the mortality of the LD group was 27.1% compared to 27.3% of the FD group [2]. A meta-analysis by Xin Qin, et al. in 2014 also came up with the same conclusion [17]. The narrow dominance of the LD group could be explained by that the higher incidence of toxicity and other side effects might influence the patients’ overall survival. Accordingly, the FD group would have greater probabilities of experiencing local or systemic side effects (including fever, sepsis and other side effects) [29], which could lead to an increase of death events. Nevertheless, an RCT published in 2005 showed that low dose group was related to a 25% of increase in recurrence rate, a 15% increase in progression rate, and a 14% increase in cancer specific death [10]. The distinct difference between this RCT and early studies and our results might due to the shorter maintenance in this study. The maintenance period of this RCT was limited to only 5–6 months which might be not sufficient according to nowadays guideline. Therefore, we assumed that the LD group has a better but not apparent efficacy than the FD group, only when the maintenance time was at least 1 year. The results of this superiority in LD group needed further validation.

The Markov probability analysis curves revealed the distribution of study populations in each state and its tendency throughout the whole course. Comparing the two curves showed in Fig. 2, the percentage of patients in the state of tumor free after TUR in the FD group was lower than that in the LD group, while the mortality went otherwise. This could be a sign that bladder cancer as a rather low-risk tumor, the recurrence without worsening of the disease did not impact much on the overall survival, which demonstrated the rationality to treat a recurrence NMIBC patient (without worsening of the disease after BCG treatment) the same way as a primary patient. However, unlike the radical feature of all RC surgery, TUR surgery for tumor recurrence requires repeated performances, causing multiple times of medical costs. In the Markov cohort simulation, 2602 times of TUR surgeries were supposed to be performed among 10,000 patients in the FD group during 20 years, comparing to 3960 times in the LD group. With reduced costs by using lower dose of BCG, but increased costs by repeating TUR, the cost-effect preponderance of adjuvant dose of BCG seems to be doubtful.

The tornado diagrams and the one-way sensitivity analysis also suggested a similar conclusion. According to the tornado diagrams, the most two effective factors in both groups were the age-specific mortality and the metastatic rate after undergoing RC, which is reasonable because these two rates were for patients about to translate to death stage or a considerably high death rate stage. The following effective factor was the rate of disease recurrence with worsening of the disease, same in the LD group and the FD group. One-way sensitivity analyses showed crossover points only in these two rates, which again revealed that it is the worsening of the disease that determine the outcomes of NMIBC rather than the disease recurrence. Nevertheless, a two-way analysis was also performed, indicating that a full-dose BCG would be a slightly dominant strategy when the rates of disease recurrence with worsening of the disease were the same in both groups. In summary, the overall survival of NMIBC patients was mainly affected by a worsening of disease.

There still exist several limitations to this study. Firstly and inevitably, the paucity of included studies might cause a bias on the transition probabilities in the model. The rate of metastasis after undergoing RC and the mortality of metastasis state were calculated from data of only two studies respectively. Secondly, we hypothesized that all the patients experienced disease recurrence without worsening of the disease would take a TUR surgery and a following BCG treatment. However, clinically, a RC might be recommended after several times of recurrence. Furthermore, neoadjuvant chemotherapy and adjuvant chemotherapy of the RC may affect the survival of the patients. Focusing on the dose comparison of NMIBC, the difference of treatment in MIBC was not discussed in this study. Additionally, according to the published studies, multiple tumors and high grades of tumors were supposed to be factors indicating a poor efficacy of BCG treatments [2, 10, 30]. This study took all intermediate and high-risk NMIBC patients into the analysis pool without a stratification analysis. Thus, on the basis of this study, a more detailed analysis taking these factors into consideration is required.

## Conclusion

Based on our efficacy analysis, the overall survivals were slightly better in the low-dose BCG treatment group than the full-dose BCG treatment group for patients with intermediate and high-risk NMIBC after undergoing TUR surgery. This finding suggested that the low-dose BCG treatment could be effectively used in NMIBC patients. However, patients in the low-dose group tended to experience a higher probability of repeating TUR surgeries, which questions the cost advantages of low-dose BCG treatment. Our findings will require further high-quality studies to validate.

## Additional file

Additional file 1: **Figure S1.** One-way sensitivity analysis of the rate of disease recurrence with disease worsening in the FD group (a) and the LD group (b). The line above represents the therapy has a better expected value. The rate of the crossover point in (a) is 0.0271, and the crossover point in (b) is 0.0371. (DOCX 11978 kb)

## Abbreviations

BCG: Bacillus calmette-guérin
FD: Full-dose
LD: Low-dose
NMIBC: Non-muscle invasive bladder cancer
RC: Radical cystectomy
RCT: Randomized controlled trial
TUR: Transurethral bladder resection

## References

[1] Babjuk M, Oosterlinck W, Sylvester R, Kaasinen E, Böhle A, Palou-Redorta J (2011). EAU guidelines on non-muscle-invasive urothelial carcinoma of the bladder, the 2011 update. Eur Urol.

[2] Oddens J, Brausi M, Sylvester R, Bono A, van de Beek C, van Andel G (2013). Final results of an eORTC-gU cancers group randomized study of maintenance bacillus calmette-guérin in intermediate- and high-risk ta, t1 papillary carcinoma of the urinary bladder: one-third dose versus full dose and 1 year versus 3 years of maintenance. Eur Urol.

[3] Shahin O, Thalmann GN, Rentsch C, Mazzucchelli L, Studer UE (2003). A retrospective analysis of 153 patients treated with or without intravesical bacillus calmette-guerin for primary stage t1 grade 3 bladder cancer: recurrence, progression and survival. J Urol.

[4] Hemdan T, Johansson R, Jahnson S, Hellström P, Tasdemir I, Malmström PU (2014). 5-year outcome of a randomized prospective study comparing bacillus calmette-guérin with epirubicin and interferon-α2b in patients with t1 bladder cancer. J Urol.

[5] Jiang S-J, Ye L-Y, Meng F-H (2016). Comparison of intravesical bacillus calmette-guerin and mitomycin c administration for non-muscle invasive bladder cancer: a meta-analysis and systematic review. Oncol Lett.

[6] Botteman MF, Pashos CL, Redaelli A, Laskin B, Hauser R (2003). The health economics of bladder cancer: a comprehensive review of the published literature. PharmacoEconomics.

[7] Lamm DL, Blumenstein BA, Crissman JD, Montie JE, Gottesman JE, Lowe BA (2000). Maintenance bacillus calmette-guerin immunotherapy for recurrent tA, t1 and carcinoma in situ transitional cell carcinoma of the bladder: a randomized southwest oncology group study. J Urol.

[8] Cheng CW, Ng MT, Chan SY, Sun WH (2004). Low dose bCG as adjuvant therapy for superficial bladder cancer and literature review. ANZ J Surg.

[9] Irie A, Uchida T, Yamashita H, Matsumoto K, Satoh T, Koh H (2003). Sufficient prophylactic efficacy with minor adverse effects by intravesical instillation of low-dose bacillus calmette-guérin for superficial bladder cancer recurrence. International journal of urology: official journal of the Japanese Urological Association.

[10] Martinez Piñeiro JA, Martinez-Piñeiro L, Solsona E, Rodríguez RH, Gómez JM, Martín MG (2005). Has a 3-fold decreased dose of bacillus calmette-guerin the same efficacy against recurrences and progression of t1G3 and tis bladder tumors than the standard dose? Results of a prospective randomized trial. J Urol.

[11] Yoneyama T, Ohyama C, Imai A, Ishimura H, Hagisawa S, Iwabuchi I (2008). Low-dose instillation therapy with bacille calmette-guérin Tokyo 172 strain after transurethral resection: historical cohort study. Urology.

[12] Mack D, Frick J (1995). Low-dose bacille calmette-guérin (bCG) therapy in superficial high-risk bladder cancer: a phase iI study with the bCG strain connaught Canada. Br J Urol.

[13] Ojea Antonio, Nogueira José Luís, Solsona Eduardo, Flores Nicolás, Gómez Jesús María Fernández, Molina Jesús Rodríguez, Chantada Venancio, Camacho José Emilio, Piñeiro Luís Martínez, Rodríguez Rafael Hernandez, Isorna Santiago, Blas Miguel, Martínez-Piñeiro José A., Madero Rosario (2007). A Multicentre, Randomised Prospective Trial Comparing Three Intravesical Adjuvant Therapies for Intermediate-Risk Superficial Bladder Cancer: Low-Dose Bacillus Calmette-Guerin (27 mg) versus Very Low-Dose Bacillus Calmette-Guerin (13.5 mg) versus Mitomycin C. European Urology.

[14] Babjuk Marko, Burger Maximilian, Zigeuner Richard, Shariat Shahrokh F., van Rhijn Bas W.G., Compérat Eva, Sylvester Richard J., Kaasinen Eero, Böhle Andreas, Palou Redorta Joan, Rouprêt Morgan (2013). EAU Guidelines on Non–Muscle-invasive Urothelial Carcinoma of the Bladder: Update 2013. European Urology.

[15] Zhu S, Tang Y, Li K, Shang Z, Jiang N, Nian X (2013). Optimal schedule of bacillus calmette-guerin for non-muscle-invasive bladder cancer: a meta-analysis of comparative studies. BMC Cancer.

[16] Astram A, Khadijah A, Yuri P, Zulfan A, Mochtar CA, Danarto R (2014). Effective dose and adverse effects of maintenance bacillus calmette-gue&apos;Rin in intermediate and high risk non-muscle invasive bladder cancer: a meta-analysis of randomized clinical trial. Acta medica Indonesiana.

[17] Qin X, Wu K, Xie L, Zhao S, Lu Y (2014). Reduced dose of bacillus calmette-guérin versus full dose of bacillus calmette-guérin for non-muscle-invasive bladder cancer after transurethral resection bladder tumor: a meta-analysis of randomized controlled trials. Chin Med J.

[18] Zeng S, Yu X, Ma C, Zhang Z, Song R, Chen X (2015). Low-dose versus standard dose of bacillus calmette-guerin in the treatment of nonmuscle invasive bladder cancer: a systematic review and meta-analysis. Medicine.

[19] Al Hussein Al Awamlh B, Lee R, Chughtai B, Donat SM, Sandhu JS, Herr HW (2015). A cost-effectiveness analysis of management of low-risk non-muscle-invasive bladder cancer using office-based fulguration. Urology.

[20] Bachir BG, Dragomir A, Aprikian AG, Tanguay S, Fairey A, Kulkarni GS (2014). Contemporary cost-effectiveness analysis comparing sequential bacillus calmette-guerin and electromotive mitomycin versus bacillus calmette-guerin alone for patients with high-risk non-muscle-invasive bladder cancer. Cancer.

[21] Kulkarni GS, Finelli A, Fleshner NE, Jewett MAS, Lopushinsky SR, Alibhai SMH (2007). Optimal management of high-risk t1G3 bladder cancer: a decision analysis. PLoS Med.

[22] Zhang Y, Denton BT, Nielsen ME (2013). Comparison of surveillance strategies for low-risk bladder cancer patients. Medical decision making : an international journal of the Society for Medical Decision Making.

[23] Naimark DMJ, Bott M, Krahn M (2008). The half-cycle correction explained: two alternative pedagogical approaches. Medical decision making : an international journal of the Society for Medical Decision Making.

[24] Beck JR, Kassirer JP, Pauker SG (1982). A convenient approximation of life expectancy (the "DEALE"). i. Validation of the method. Am J Med.

[25] Pfister C, Kerkeni W, Rigaud J, Le Gal S, Saint F, Colombel M (2014). Efficacy and tolerance of one-third full dose bacillus calmette-guérin maintenance therapy every 3 months or 6 months: two-year results of uRO-bCG-4 multicenter study. International journal of urology: official journal of the Japanese Urological Association.

[26] Martinez-Piñeiro L, Portillo JA, Fernandez JM, Zabala JA, Cadierno I, Moyano JL (2015). Maintenance therapy with 3-monthly bacillus calmette-guerin for 3 years is not superior to standard induction therapy in high-risk non–muscle-invasive urothelial bladder carcinoma: final results of randomised cUETO study 98013. Eur Urol.

[27] Heron M, Hoyert DL, Murphy SL, Xu J, Kochanek KD, Tejada-Vera B (2009). Deaths: final data for 2006. National vital statistics reports: from the Centers for Disease Control and Prevention, National Center for Health Statistics, National Vital Statistics System.

[28] Sonnenberg FA, Beck JR (1993). Markov models in medical decision making: a practical guide. Medical decision making: an international journal of the Society for Medical Decision Making.

[29] Brausi M, Oddens J, Sylvester R, Bono A, van de Beek C, van Andel G (2014). Side effects of bacillus calmette-guérin (bCG) in the treatment of intermediate- and high-risk ta, t1 papillary carcinoma of the bladder: results of the eORTC genito-urinary cancers group randomised phase 3 study comparing one-third dose with full dose and 1 year with 3 years of maintenance BCG. Eur Urol.

[30] Martínez-Piñeiro JA, Flores N, Isorna S, Solsona E, Sebastián JL, Pertusa C (2002). Long-term follow-up of a randomized prospective trial comparing a standard 81 mg dose of intravesical bacille calmette-guérin with a reduced dose of 27 mg in superficial bladder cancer. BJU Int.

[31] Aziz A, May M, Burger M, Palisaar RJ, Trinh QD, Fritsche HM (2014). Prediction of 90-day mortality after radical cystectomy for bladder cancer in a prospective european multicenter cohort. Eur Urol.

[32] Amling CL, Thrasher JB, Frazier HA, Dodge RK, Robertson JE, Paulson DF (1994). Radical cystectomy for stages ta, tis and t1 transitional cell carcinoma of the bladder. J Urol.

[33] Leveridge MJ, Siemens DR, Mackillop WJ, Peng Y, Tannock IF, Berman DM (2015). Radical cystectomy and adjuvant chemotherapy for bladder cancer in the elderly: a population-based study. Urology.

[34] Hounsome LS, Verne J, McGrath JS, Gillatt DA (2015). Trends in operative caseload and mortality rates after radical cystectomy for bladder cancer in England for 1998-2010. Eur Urol.

[35] Spaliviero M, Dalbagni G, Bochner BH, Poon BY, Huang H, Al-Ahmadie HA (2014). Clinical outcome of patients with t1 micropapillary urothelial carcinoma of the bladder. J Urol.

[36] Koie T, Ohyama C, Yamamoto H, Imai A, Hatakeyama S, Yoneyama T, et al. Differences in the recurrence pattern after neoadjuvant chemotherapy compared to surgery alone in patients with muscle-invasive bladder cancer. Med Oncol (Northwood, London, England) 2015; 32:421. 10.1007/s12032-014-0421-x 25471790

[37] von der Maase H, Hansen SW, Roberts JT, Dogliotti L, Oliver T, Moore MJ (2000). Gemcitabine and cisplatin versus methotrexate, vinblastine, doxorubicin, and cisplatin in advanced or metastatic bladder cancer: results of a large, randomized, multinational, multicenter, phase III study. J Clin Oncol Off J Am Soc Clin Oncol.

[38] Kaufman DS, Shipley WU, Feldman AS (2009). Bladder cancer. Lancet.

