# Supplementary material for: Low-dose Bacillus Calmette-Guerin versus full-dose for intermediate and high-risk of non-muscle invasive bladder cancer: a Markov model
Source: BMC Cancer. 2018 Nov 12;18:1108. doi: 10.1186/s12885-018-4988-z (PMC6233591; doi:10.1186/s12885-018-4988-z)
Supplement: Supplementary file 1 — Figure S1. One-way sensitivity analysis of the rate of disease recurrence with disease worsening in the FD group (a) and the LD group (b). The line above represents the therapy has a better expected value. The rate of the crossover point in (a) is 0.0271, and the crossover point in (b) is 0.0371. (DOCX 11978 kb) [file 12885_2018_4988_MOESM1_ESM.docx]

Low-dose Bacillus Calmette-Guerin versus Full-dose for Intermediate and High-risk of Non-muscle Invasive Bladder Cancer: A Markov Model

Zongren Wang^1,*^, Han Xiao^2,*^, Guangyan Wei^3, *^, Ning Zhang^2^, Mengchao Wei^3^, Zebin Chen^3^, Zhenwei Peng^4,5^, Sui Peng^4^, Shaopeng Qiu^1^, Heping Li^5,#^ , Jianting Long^5,#^

^1^ Department of Urology, The First Affiliated Hospital of Sun Yat-Sen University, Guangzhou, Guangdong, China.

^2^ Department of Gastroenterology and Hepatology, The First Affiliated Hospital of Sun Yat-Sen University, Guangzhou, Guangdong, China.

^3^ Department of Liver Surgery, The First Affiliated Hospital of Sun Yat-sen University, Guangzhou, Guangdong, China.

^4^ Department of clinical trials center, The First Affiliated Hospital of Sun Yat-Sen University, Guangzhou, Guangdong, China.

^5^ Department of Oncology, The First Affiliated Hospital of Sun Yat-sen University, Guangzhou, Guangdong, China

* These authors contribute equally to the study.


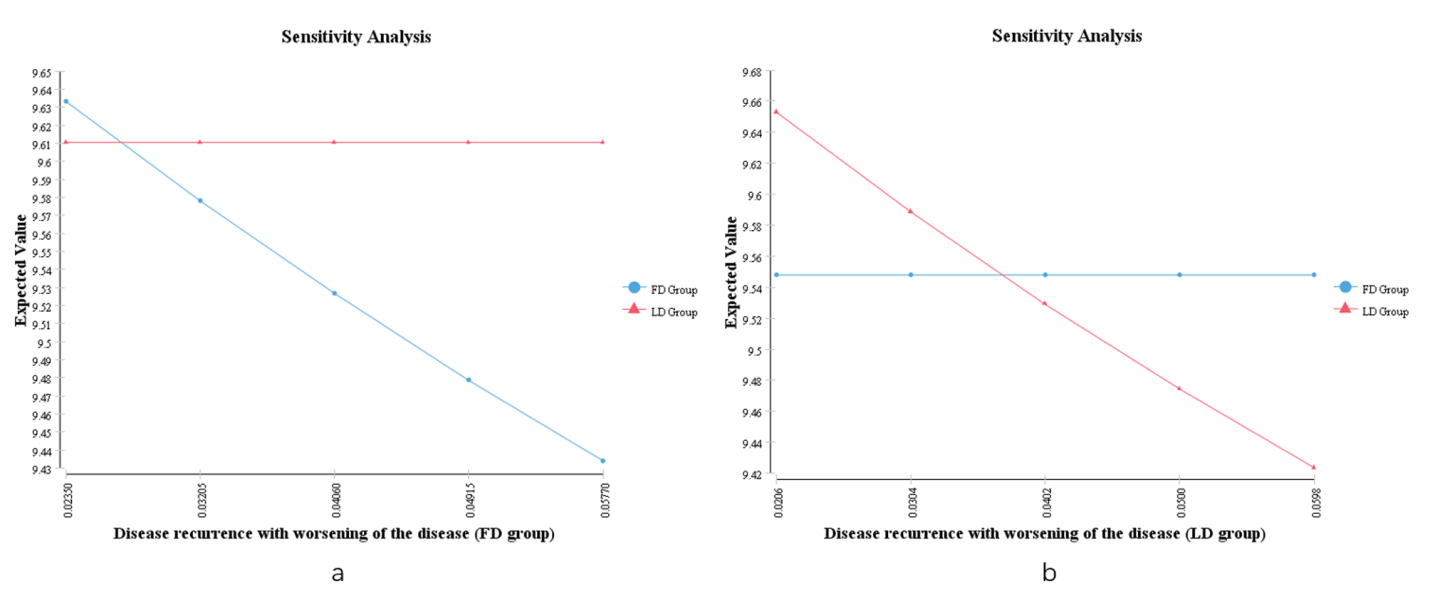


**Supplementary Figure 1.** One-way sensitivity analysis of the rate of disease recurrence with disease worsening in the FD group (a) and the LD group (b). The line above represents the therapy has a better expected value. The rate of the crossover point in (a) is 0.0271, and the crossover point in (b) is 0.0371.


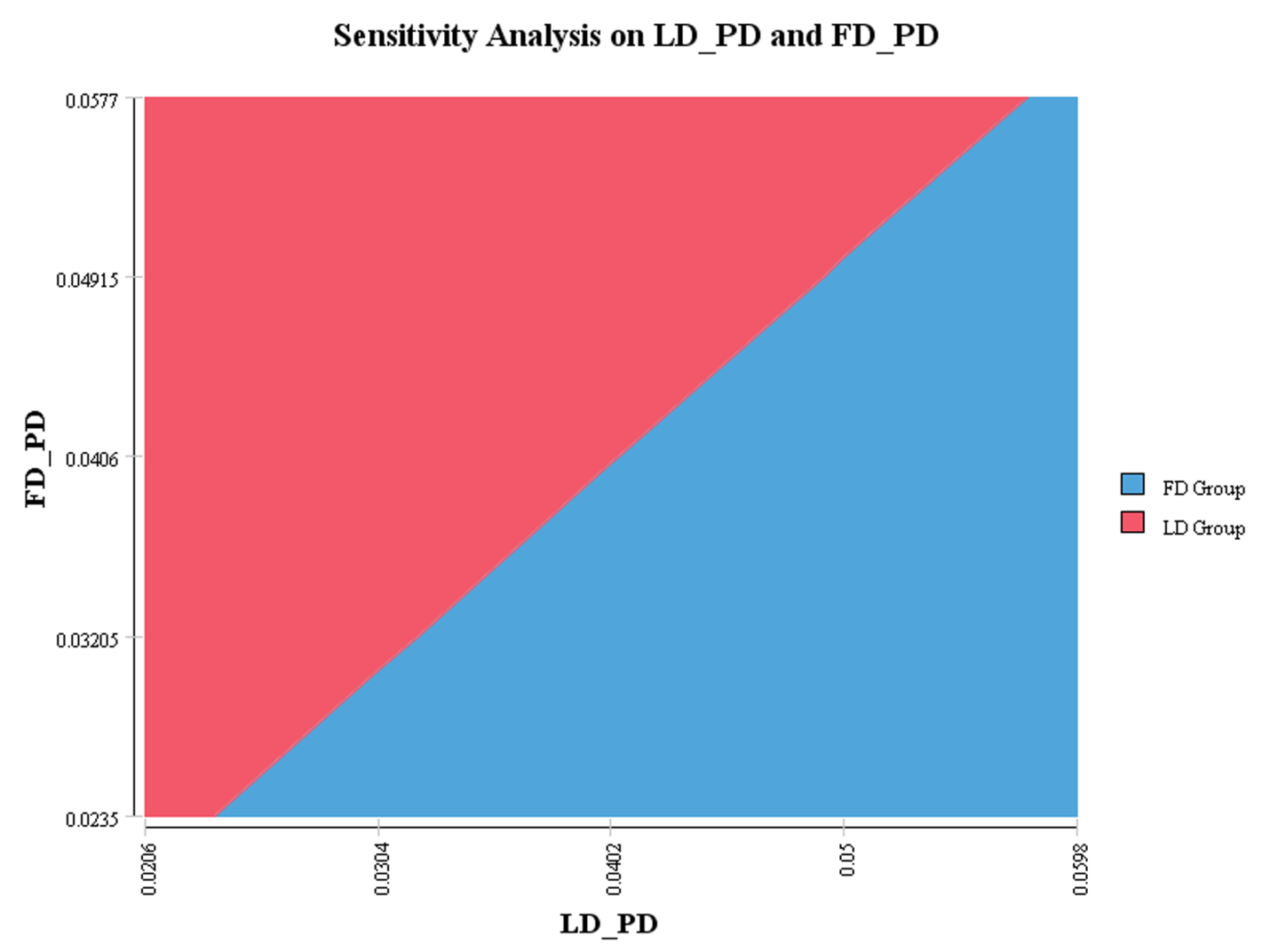


**Supplementary Figure 2.** Two-way sensitivity analysis of the rates of disease recurrence with disease worsening in the FD group and the LD group. FD_PD stands for the rate of disease recurrence with worsening of the disease(FD group), and LD_PD stands for the rate of disease recurrence with worsening of the disease(LD group). Points in the blue area indicate that a full-dose BCG treatment would have a better overall survival in this situation, while points in the red area represent the opposite. Abbreviations: FD_PD, rate of disease recurrence with worsening of the disease (FD group); LD_PD, rate of disease recurrence with worsening of the disease (LD group).

Supplementary Material: Analysis of the Markov model with a 3-year maintenance period

A Markov model with a 3-year maintenance period was constructed. The cycle-length was 3 years. The follow-up duration was set to be 7 cycles, which was 21 years. Other details of model construction were the same to the model we describe in the main text. The expected overall survivals were 9.78 years for the FD group and 9.84 years for the LD group. The Markov probability analysis curves were showed in **Supplementary Figure 3**. The survival curves were showed in **Supplementary Figure 4**.

Tornado diagrams analyzing the two therapies were showed in **Supplementary Figure 5**. One-way analysis showed that the rate of disease recurrence with worsening of the disease seemed to affect the efficacy superiority between the two groups. The FD group would have a better efficacy if this rate of FD group was less than 7.9%, or the rate of LD group was over 10.7% (**Supplementary Figure 6**). All other parameters showed no effect on the superiority of LD group.

Details of a Monte Carlo simulation with the sample of 10000 patients for each therapy,were showed in **Supplementary Figure 7.** The median survival of the FD group was 9.81 years compared to 9.87 years for the LD group. The 95%CI of overall survival in the FD group was (9.35-10.26) years, while that in the LD group was (9.41-10.33) years (*P*<0.001), indicating a better but not apparent (compared to the expected OS of NMIBC patients) outcome in the LD group.


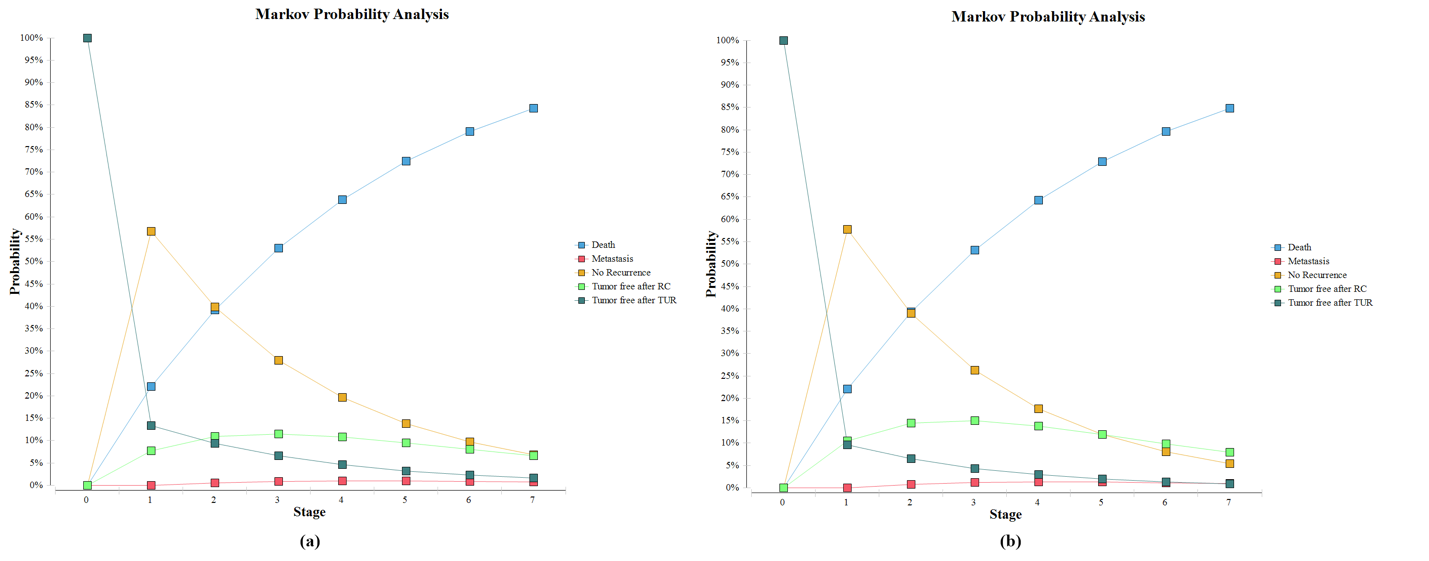


**Supplementary Figure 3.** Markov probability analyses for the LD group (a) and the FD group (b). These diagrams showed the details of the distribution of the patients in each stage. Abbreviations: FD, full-dose; LD, low-does.


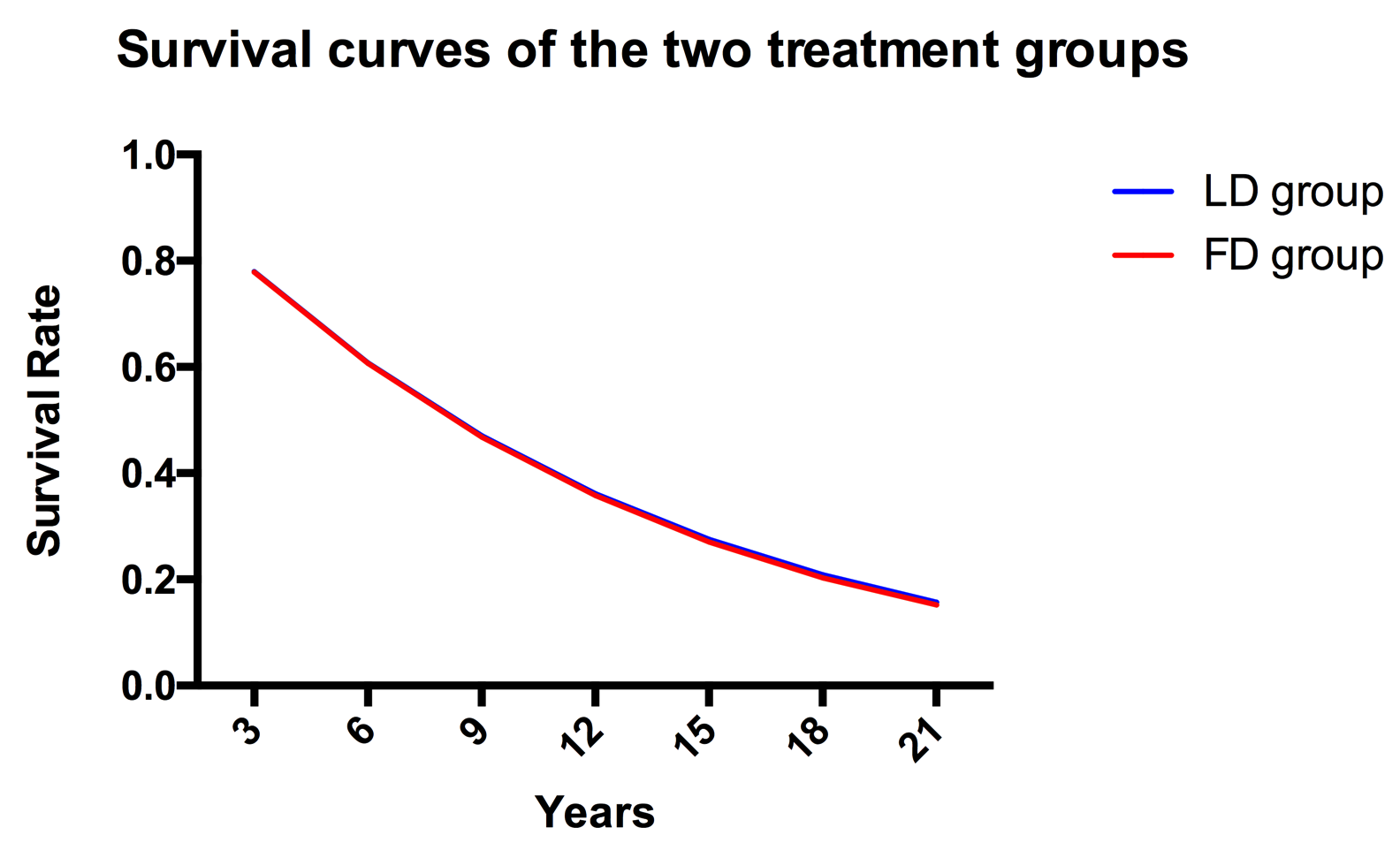


**Supplementary Figure 4.** Survival curves of the two treatment groups. The two curves representing the survival rates of the FD group and the LD group were highly coincident, indicating that the priority of the LD group was not apparent. Abbreviations: FD, full-dose; LD, low-does.


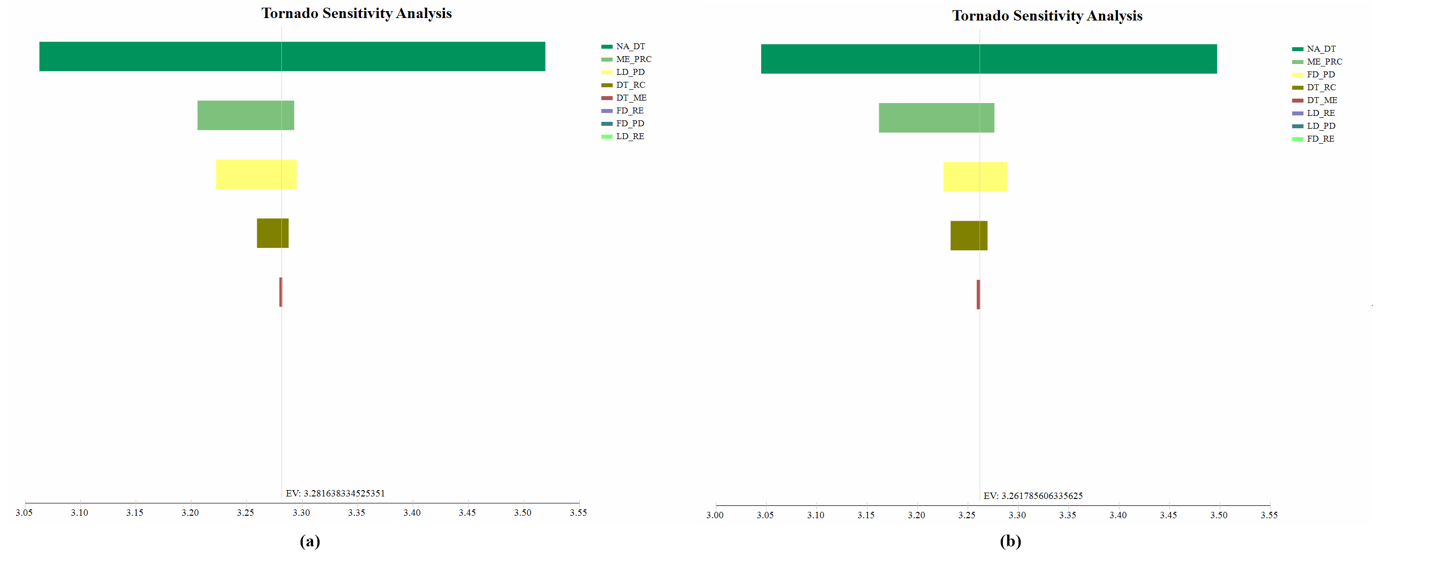


**Supplementary Figure 5.** Tornado diagrams for the LD group (a) and the FD group (b). Tornado diagrams analyzed all the parameters in this model. The length of colored bar for each parameter represents the extent of its effect on the overall survival. The longer a bar is, the larger its effect is. Abbreviations: NA_DT, age-specific mortality; ME_PRC, metastatic rate after underwent RC; FD_PD, rate of disease recurrence with worsening of the disease(FD group); DT_RC, mortality of RC surgery; DT_ME, death rate of metastatic state; LD_RE, rate of disease recurrence without worsening of the disease (LD group); LD_PD, rate of disease recurrence with worsening of the disease(LD group); FD_RE, rate of disease recurrence without worsening of the disease(FD group).


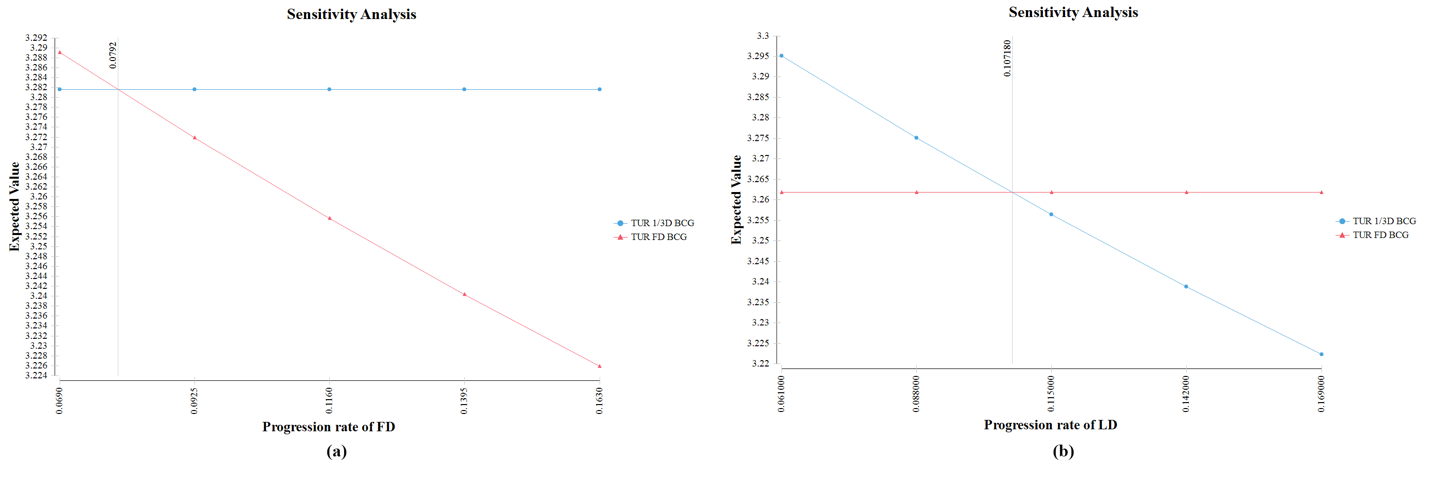


**Supplementary Figure 6.** One-way sensitivity analysis of the rate of disease recurrence with disease worsening in the LD group (a) and the FD group (b). The line above represents the therapy has a better expected value. The rate of the crossover point in (a) is 0.0792, and the crossover point in (b) is 0.10719.


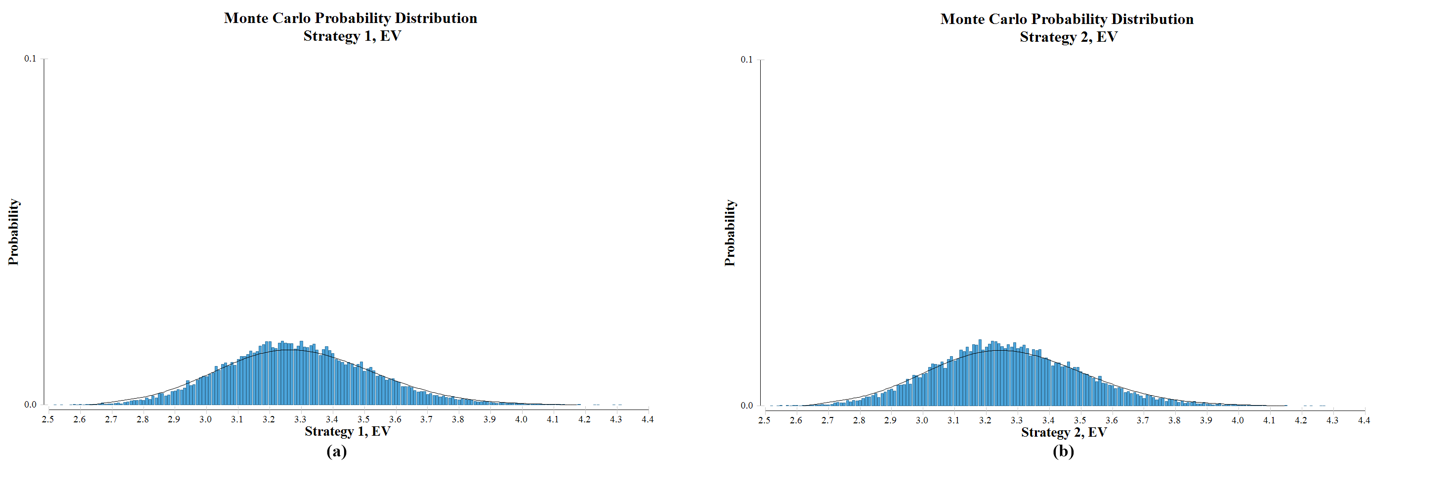


**Supplementary Figure 7.** The second-order Monte Carlo probability distribution of the overall survival in the LD group (a) and the FD group (b). EV stands for expected value, which is the overall survival in this situation. The length of the bar represents the probability of an expected value in the 10000 population. Abbreviations: EV, expected value.
